# Bell's Palsy: Symptoms Preceding and Accompanying the Facial Paresis

**DOI:** 10.1155/2014/801971

**Published:** 2014-11-27

**Authors:** Daniele De Seta, Patrizia Mancini, Antonio Minni, Luca Prosperini, Elio De Seta, Giuseppe Attanasio, Edoardo Covelli, Andrea De Carlo, Roberto Filipo

**Affiliations:** ^1^Department of Sense Organs, Sapienza University of Rome, Policlinico Umberto I, Viale del Policlinico 155, 00161 Rome, Italy; ^2^Department of Neurology, Sapienza University of Rome, Italy

## Abstract

This individual prospective cohort study aims to report and analyze the symptoms preceding and accompanying the facial paresis in Bell's palsy (BP). Two hundred sixty-nine patients affected by BP with a maximum delay of 48 hours from the onset were enrolled in the study. The evolution of the facial paresis expressed as House-Brackmann grade in the first 10 days and its correlation with symptoms were analyzed. At the onset, 136 patients presented postauricular pain, 114 were affected by dry eye, and 94 reported dysgeusia. Dry mouth was present in 54 patients (19.7%), facial pain, hyperlacrimation, aural fullness, and hyperacusis represented a smaller percentage of the reported symptoms. After 10 days, 39.9% of the group had a severe paresis while 10.2% reached a complete recovery. Dry mouth at the onset was correlated with severe grade of palsy and was prognostic for poor recovery in the early period. These outcomes lead to the deduction that the nervus intermedius plays an important role in the presentation of the BP and it might be responsible for most of the accompanying symptomatology of the paresis. Our findings could be of important interest to early address a BP patient to further examinations and subsequent therapy.

## 1. Introduction

Bell's palsy (BP) represents more than 70% of peripheral acute idiopathic facial paresis, widespread all over the world with an incidence in different regions ranging from 10 to 40 per 10.000 persons [1]. Although generally unilateral, it is described in some rare cases involving both facial nerves [2]. The causes of the paresis still remain unknown even if the viral etiology has been discussed by several authors, and herpes viruses seemed to be the most plausible infective agent determining inflammation and swelling of the nerve with subsequent blockage of the neural activity [3, 4]. The natural history of BP is encouraging for the patients since a total recovery of facial function is expected in 70–85% of the patients, and a higher percentage of recovery is achieved if corticosteroid therapy is administered [5, 6] and early physical rehabilitation is performed in severe grades of paresis [7].

The facial nerve (FN) is mainly a motor nerve and provides innervation to the mimic muscles of the ipsilateral half of the face; it also innervates the posterior belly of the digastric muscle, the stapedius muscle, and the stylohyoid muscle. The sensory and parasympathetic functions of FN are carried by fibers that constitute the nervus intermedius (NI). The NI, also known as Wrisberg nerve or intermediate nerve, is commonly described as a root of the FN containing sensory and parasympathetic fibers, although for some authors it is considered, since the first anatomical studies in the 18th century, as an independent nerve [8–11]. Along the NI via the chorda tympani, sensory fibers derived from the gustatory receptors travel from the anterior two-thirds of the tongue, floor of the mouth, and palate, directed to the superior pole of the solitary nucleus in the medulla. Sensory information from the skin of the external auditory canal, concha, and from the mucous membranes of the nasopharynx and palate is carried via the greater petrosal nerve that originates from the geniculate ganglion. Nerve fibers derived from the superior salivary nucleus provide innervation to the lacrimal, submandibular, and sublingual glands. Postganglionic parasympathetic fibers from pterygopalatine ganglion innervate the lacrimal gland and the mucosal glands of the nose, palate, and pharynx. Parasympathetic innervation serves to control the flow of saliva and tears from these glands.

FN paresis represents only one of the existing symptoms of BP that normally comprehends different other manifestations. The “nonparetic” symptomatology is due to NI dysfunction. Postauricular pain or taste disorders are frequently reported from the patients together with the paresis, otherwise as symptoms which occurred prior to the onset of the paresis. The role of prodromal or associated symptoms in BP has not been studied extensively, and in literature few papers report the epidemiologic aspect of these symptoms in the very early stage of the palsy and their role in the evolution of the paresis.

Preceding and associated symptoms in the first phase of Bell's palsy have been studied in patients observed and treated in our department during the last 5 years, in order to assess whether a correlation was present with the evolution and the prognosis of the disease.

## 2. Material and Methods

Between March 2008 and December 2012 all the patients affected by facial paralysis that reached the emergency department of our hospital have been evaluated. Each patient underwent neurological examination in order to exclude central nervous system involvement and microscopic examination of both ears. CT scan and/or MRI were performed when clinical history or clinical signs suggested a secondary facial palsy. All the patients that were diagnosed with a peripheral facial palsy (unless in the case of a serious contraindication to steroid therapy) were prescribed a standardized oral pharmacological treatment with prednisone 1 mg/Kg for 10 days in association with valacyclovir 500 mg 3 times/day for 6 days. The patients with incomplete eye closure with maximum effort were invited to put artificial tear drops or gel and tape the eye during the night; all those presenting inflammation/hyperemia of the eye were addressed to an ophthalmological evaluation. After the first 10 days of therapy the patients were addressed to a rehabilitation treatment in relation to their clinical condition.

The eligibility criteria to the study were as follows: adults between 15 and 70 years of age, Bell's palsy within 48 hours from onset, and no previous pharmacological therapy for the episode of facial palsy. Exclusion criteria included any sign of infective, metabolic, central, and peripheral nervous system disease, temporal bone pathologies, pregnancy, and signs and/or symptoms of varicella zoster virus (VZV) infection.

Concerning the study group, the otolaryngological examination included anamnesis of previous ipsilateral or contralateral facial weakness (recurrent palsy), family history of previous facial palsies, and close attention and recording of the onset symptom and all the other symptoms. The clinical evaluation of the BP stage was carried out via the House-Brackmann (HB) Facial Grading System chosen for its simplicity of administration, for its wide use and recognized validity, and its robustness and internal consistency.

The patients were followed up always by the same four physicians and a HB score was assigned at each control. The followup included evaluations at the end of the therapy (day 10), at 30 days from the onset, and every month for six months or until the total recovery. The present study is focused on the analysis of data collected at the day of the first visit and at the 10-day endpoint, taking into account the evolution of the disease before the enrollment in different rehabilitation groups. Indeed in the first 10 days all patients received the same treatment, reducing the possibility of bias due to different therapies, in the analysis of the disease evolution and its correlation with the debut symptoms.

All the patients signed a written informed consent; the study was approved by the Ethical Committee of Sapienza University authorization number 29-05-08/1432.

### 2.1. Statistical Analysis

Data are presented as proportion (%) and mean (standard deviation, SD) as appropriated. Correlations were tested by the Kendall Tau coefficient. A classification and regression tree-based analysis [12] was performed to define the best classification of patients by the HB grade at 10 days after the onset (i.e., the dependent variable) over all potential predictors (i.e., all the independent variables considered at first evaluation) and all possible cutpoints. The order of the variables and the specific cutpoints was detected using an exhaustive chi-squared automatic interaction detection (CHAID) algorithm that, at each node, selects those predictors having the strongest interaction with the dependent variable, after examining each possible split.

All *P* values less than 0.05 in either direction were considered significant. Analyses were carried out using a PC version of Statistical Package for Social Sciences 16.0 (SPSS, Chicago, IL, USA).

## 3. Results

Two hundred sixty-nine patients affected by BP were included in the study. The incidence of BP in our hospital was 14.7/10 000 patients/year. Male-to-female ratio was 1.29/1 and mean age 48.6 years. No significant difference has been noted between male/female or left/right side incidence of palsy.

**Figure 1:**
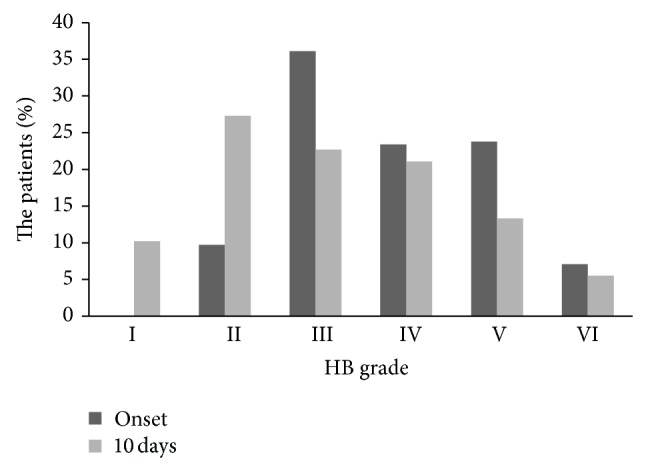
Distribution of the severity of the facial paresis according to House-Brackmann (HB) facial grading scale at the onset and after 10 days.

At the first examination the moderate-to-severe palsies (HB grades IV–VI) represented the 54.3% of the patients; at ten days from the onset these palsies represented the 39.9% whilst a complete recovery was observed in 10.2% of patients. The evolution of the grade of the paresis after the first ten days is reported in Figure 1.

At the first evaluation all the patients presented with the facial paralysis as the main symptom, apart from 3 patients, treated by our group in the previous years, who came to our observation with prodromal symptoms, before the onset of the paresis; all the three patients developed a facial paresis in the following days.

**Figure 2:**
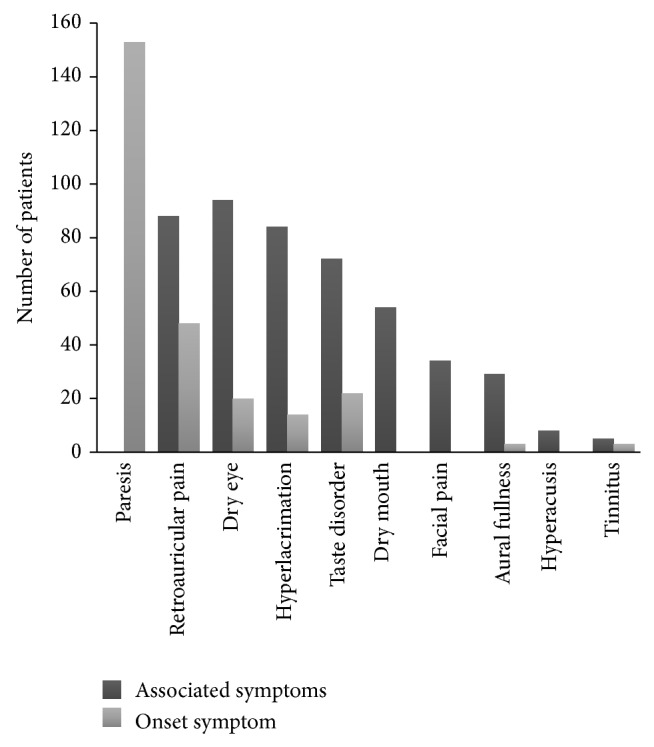
Onset symptoms and accompanying symptoms over the study group. Paresis is represented only as onset symptom since the whole number of patients will eventually develop facial numbness.

For what concerns the accompanying symptoms observed at the first visit, in 50.5% of the patients postauricular pain was present. 34.7% of the patients reported dysgeusia due to chorda tympani involvement, and the 37.9% of the total was affected by dry eye for the involvement of the greater superficial petrosal nerve. Xerostomia was present at the first visit in 19.7% of the patients; facial pain, hyperlacrimation, aural fullness, and hyperacusis represented a smaller percentage of the symptoms (Figure 2).

Taking close attention to the symptoms we asked the patients if the paresis/paralysis was the first symptom that occurred or if they noted any prodromal symptom. In more than forty percent of the patients the paresis was not the first symptom, but it was retroauricular pain in 17.5% of the patients, dysgeusia in 7.8%, dry eye in 7.1%, and hyperlacrimation in 4.8% (Figure 2). All the concurrent symptoms disappeared together with the recovery of the palsy except for the retroauricular pain that in some cases lasted longer than the palsy and was treated, when not spontaneously regressed one month after the recovery of the palsy, with administration of pregabalin for a short period of time.

Kendell Tau coefficient test showed a significant correlation of some symptoms at the onset and a higher HB grade at the onset (Table 1). Higher grades of paresis were more often associated with dry mouth, aural fullness, retroauricular pain, and taste disorder.

**Figure 3:**
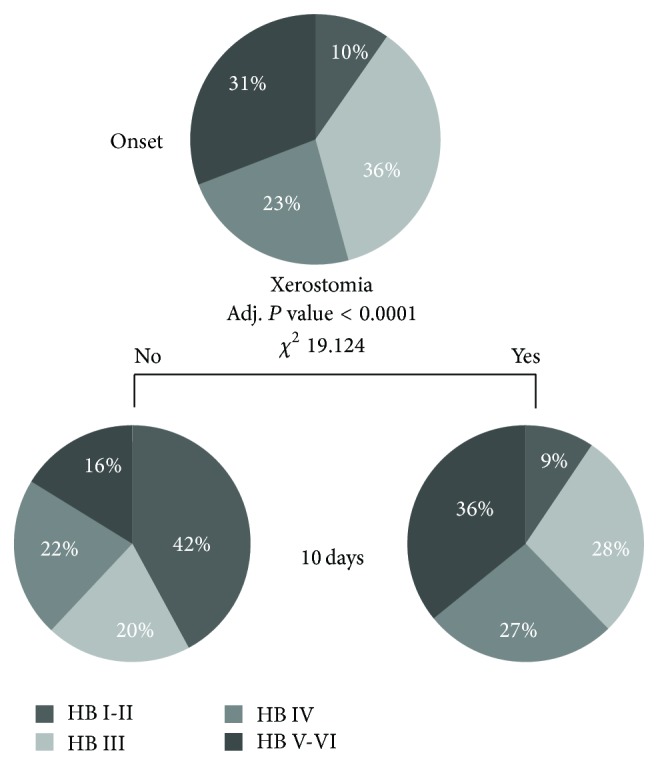
Chi-squared automatic interaction detection (CHAID) algorithm.

Figure 3 shows the tree-based classification with different severity of paresis (according to HB scale) at the onset and 10 days later. At 10 days most of the patients had an improvement of the paresis with recovery in 35.7% of the cases (HB grade I/II), 58 out of 269 patients (21.6%) had a HB grade III, 61 out of 269 (22.7%) presented with a HB grade IV, and the remaining 54 patients (20.1%) had a severe palsy (HB V/VI). Among all the variables evaluated (onset symptoms, associated symptoms, age, and sex) the presence of xerostomia or dry mouth at the first evaluation was a significant predictor of bad prognosis at 10 days (adjusted *P* value < 0.0001). The patients who complained of xerostomia as associated symptom were more likely to have a severe palsy after 10 days from BP onset. The overall risk of misclassification for the first cut point was 5.2% for HB grades II/III, indicating that the absence of xerostomia was associated with a better prognosis in the majority of patients. By contrast, the risk of misclassification was 64.8% for HB grade V/VI, indicating that only about one-third of patients with poor recovery at 10 days complained of xerostomia. Other associated symptoms did not reach a significant *P* value to be considered as cutpoints.

## 4. Discussion

Preceding symptoms in Bell's palsy have been described and reported in other studies [13–16]. To our knowledge this study represents the first accurate report of all the symptomatology that occurs in the very first phase of Bell's palsy, with an attempt to give a prognostic statistical value to these symptoms.

### 4.1. Ear Pain

The pathogenesis of pain around the ear, interesting the face or the neck in Bell's palsy, is not completely clear. Adour et al. [17] stated that the anoxia of the nerve, caused by a primary or secondary ischemia, followed by compensatory dilatation of the blood vessels supplying the nerve, is part of the process causing the occurrence of ear pain. Retroauricular pain would then result from the dilation phase of the vessels, alike the pain associated with migraine. As described above the NI has a branch carried via the greater petrosal nerve that carries sensations from the skin in the region of the external ear and mastoid region. Facial nerve inflammation involving this nerve branch would result as an ipsilateral pain in this area. The role of nervi nervorum in facial and retroauricular pain in BP has been explained by Han in a recent paper [18]. The facial nerve, alike all peripheral nerves, has free nerve endings in the perineurium and endoneurium, derived from fibres in the nerve trunk itself, which have nociceptive function [19]. When a peripheral nerve is damaged, three types of pain can be produced. First, nerve trunk pain is described as aching, knifelike, or tender and is attributed to increased activity in mechanically or chemically sensitized nociceptors within the nerve sheath. Second, dysesthetic pain is described as burning, tingling, searing, or electric and attributed to damaged nociceptive afferent axons themselves. Third, there can be referred pain from the nerve sheaths innervated by nervi nervorum through central convergence. Thus the facial pain as well as the pain at nerve exit from the stylomastoid foramen would be explained by the involvement nerve trunk nociceptors, while the pain in the retroauricular region and the neck would be generated by algogenic stimuli from the sheaths of facial nerve delivered to trigeminocervical nuclear complex via nervi nervorum.

Retroauricular pain preceding the onset of the paralysis has been described by other studies [13–15]. In our study retroauricular pain was present in 50.5% of the whole study group and preceded the onset of the palsy in 17.5% of cases; these findings are in accordance with the percentages reported by the other authors. In this study retroauricular pain as an accompanying as well as a preceding symptom had no prognostic significance for the severity of the palsy at 10 days, in accordance with Chida et al., Berg et al., and Adour et al. [15–17]; the Swedish group stated that the pain persisting for 2 weeks or presenting between 11 and 17 days from the onset of the paresis has a negative prognostic factor. Other studies found ear pain as a risk factor for incomplete recovery [13, 20, 21].

### 4.2. Tearing Problems

Lacrimation disorders are common both in the first phase of Bell's palsy and in the sequelae. Efferent fibres of the NI from the superior salivary nucleus reach the lacrimal gland through the postganglionic parasympathetic fibres originated from the pterygopalatine ganglion. The effect of the FN/NI paresis would be, according to Peitersen, the reduction of the gland's secretion while the hyperlacrimation is caused by the paralytic lagophthalmos that impede tears to be transported medially to the lacrimal sac. Nevertheless the percentage of our patients complaining of increased tearing is reported in Figure 2. On the other hand a total of 42% of our patients complained of dry eye. In the study of Peitersen the author reports 11% of reduced or abolished tearing after the lacrimation test; the difference between this and our findings could be related to a sensation of dry eye reported by the patients but actually caused by the impossibility to have an efficient blink reflex or by the different timing of the first evaluation being in our study in the very early period. Kawamoto and Ikeda studied the greater petrosal nerve function in BP/Ramsay Hunt syndrome patients by means of Schirmer's tear test and soft palate electrogustometry (EGM) [22]; in their study EGM appears to be a more sensitive method for testing the function of the GPN; the results of lacrimal function test were more closely related to the severity of and prognosis for facial paralysis than the results of EGM.

### 4.3. Taste Disorder

Chorda tympani involvement during Bell's palsy is very common and altered taste is one of the most frequently reported symptoms in a patient affected by idiopathic facial paresis. Taste disorder was present in about a third of our study group (34.9%); the present data completely agrees with those elsewhere reported [13, 22], although when examined objectively, 83% of patients had a reduced or abolished taste sensation in the ipsilateral half tongue [13]. The difference can be explained by the fact that patients can still use the normal side of the tongue for the taste function. Taste disorder preceding the onset of the paresis was reported by 22 patients (8.1%) highlighting the heterogeneous onset manifestation of Bell's palsy.

### 4.4. Salivary Problems

Parasympathetic fibres originating from the superior salivary nucleus carried by the chorda tympani provide the innervation to the submandibular and sublingual glands. Few papers have studied the salivary flow in BP [14, 23]. Ekstrand, over 239 patients affected by BP, found the sialometry to be a reliable prognostic examination to identify patients with good or poor facial outcomes at 12 months, when a powerful secretory stimulus is used. In our group 20% of patients complained of a dry mouth and this record was found to be correlated with severe facial paralysis at the onset (*P* = 0.001) and was found to be prognostic for a severe grade of paralysis at 10 days (*P* < 0.0001). Alfieri et al. studied the salivary flow and the acute phase proteins in both extraparotid and parotid saliva and found a reduced salivary flow rate from the parotid gland on the paralysed side compared to the healthy side, without reaching a statistically significant value; nevertheless, the role of the intermediate nerve in parasympathetic innervation of the parotid gland is not clear and besides a direct innervation route is not known [24].

### 4.5. Aural Fullness

Although aural fullness is not a typical symptom described in BP and has never been reported so far, 11.8% of our patients complained of “ear pressure” or a “clogging sensation” of the ear ipsilateral to the paresis. Arnold's nerve is formed by a large main branch from the superior ganglion of the 10th cranial nerve and a small branch from the inferior ganglion of the 9th cranial nerve; passing within a small bony channel in the fallopian canal it divides into two branches. The superior branch gives off twigs that appear to end in the facial nerve sheath; the inferior branch is joined by a twig from facial/intermediate nerve and continues in a bony channel to provide cutaneous sensation to posterior surface of the external auditory canal [25]. Arnold's nerve involvement in acoustic neuroma patients is responsible for the Hitselberger sign [26], described as external auditory canal hypoesthesia; in the same way its involvement in BP could be responsible for the sensation of pressure in the ear described by our patients.

## 5. Conclusion

Facial paresis in Bell's palsy is a symptom of more complex and heterogeneous pathology that could begin with the facial paresis itself or with one of the other symptoms. The NI involvement plays an important role in the clinical presentation of the BP and it is responsible for most of the accompanying symptomatology of the paresis. Dry mouth at the onset is highly correlated with a severe grade of palsy, and when present it is related to a poor chance of recovery in the early period. This finding could be of important interest for those centers where the access to the electrophysiological testing is not easy and where the choice of referring BP patients to further examinations (EMG, EnoG, Blink Reflex) [27] and subsequent therapy (physical rehabilitation and surgical decompression) is made more difficult. This study investigated the symptoms on the basis of the patient's history only without the use of objective examinations; nevertheless, a correlation with data present in literature was found. The association of these results with those coming from the electrophysiological tests will be object of further study in order to better evaluate the BP patients in the first days after the onset and address them to the most appropriate therapy.

## Figures and Tables

**Table 1:** Severity of facial palsy and symptomatology.

Symptom	Correlation coefficient	*P* value
Dysgeusia	−0.137	0.014
Retroauricular pain	0.156	0.005
Aural fullness	0.168	0.002
Xerostomia	0.0191	0.001

Correlation between symptoms and severity of facial paralysis at the onset according to the Kendall Tau coefficient test.
